# Identification, selection, and expansion of non-gene modified alloantigen-reactive Tregs for clinical therapeutic use

**DOI:** 10.1016/j.cellimm.2020.104214

**Published:** 2020-11

**Authors:** Alaa Alzhrani, Matthew Bottomley, Kathryn Wood, Joanna Hester, Fadi Issa

**Affiliations:** Transplantation Research Immunology Group, Nuffield Department of Surgical Sciences, University of Oxford, Oxford OX3 9DU, United Kingdom

**Keywords:** APC, antigen presenting cells, arTreg, alloantigen reactive regulatory T cell, CAR, chimeric antigen receptor, CTLA-4, cytotoxic T-lymphocyte antigen 4, DC, dendritic cell, FACS, fluorescence-activated cell sorting, GMP, good manufacturing practice, GVHD, graft vs host disease, HLA, human leukocyte antigen, iDCs, immature DCs, IDO, indoleamine 2,3-dioxygenase, MDSCs, myeloid-derived suppressor cells, Mregs, regulatory macrophages, polyTregs, polyclonally-expanded Tregs, pTreg, peripheral regulatory T cell, Tconv, conventional T cell, TCR, *T*-cell receptor, TCR-Treg, TCR-transduced Treg, Treg, regulatory T cell, Tr1, type 1 regulatory cells, TSDR, regulatory T cell-specific demethylated region, tTreg, thymus-derived regulatory T cell, Alloantigen-reactive Tregs, Cellular therapy, Clinical trials, polyTregs, Regulatory T cells, Transplantation

## Abstract

Transplantation is limited by the need for life-long pharmacological immunosuppression, which carries significant morbidity and mortality. Regulatory T cell (Treg) therapy holds significant promise as a strategy to facilitate immunosuppression minimization. Polyclonal Treg therapy has been assessed in a number of Phase I/II clinical trials in both solid organ and hematopoietic transplantation. Attention is now shifting towards the production of alloantigen-reactive Tregs (arTregs) through co-culture with donor antigen. These allospecific cells harbour potent suppressive function and yet their specificity implies a theoretical reduction in off-target effects. This review will cover the progress in the development of arTregs including their potential application for clinical use in transplantation, the knowledge gained so far from clinical trials of Tregs in transplant patients, and future directions for Treg therapy.

## Highlights

• Tregs are a major focus of investigation in transplantation for immunosuppression minimisation.
• Development of alloantigen-specific Tregs has the potential to improve efficacy and safety.
• A number of methodologies for production exist including culture with donor alloantigen.
• Clinical trials of polyclonal Treg therapy are now moving into Phase II and beyond.

## 1 Introduction

Transplantation is limited by the inability to control graft alloresponses specifically and the consequent need for life-long global pharmacological immunosuppression. These immunosuppressive drugs contribute to significant morbidity and mortality arising from their off-target effects, which include life-threatening infection, cardiovascular disease, metabolic disorders, and malignancy [1], [2]. Moreover, immunosuppression itself may be directly toxic to the organ transplant and therefore contribute to poor long-term outcomes.

Given these challenges, there has been significant attention in the past few decades from the transplant community to develop therapeutic strategies that facilitate the minimization or even cessation of pharmaceutical immunosuppression. In particular, there is focus on cellular therapy, which could naturally and specifically regulate the alloimmune response. Among these cell therapies, the canonical CD4^+^ Tregs are the most understood and closest to clinical adoption [3]. In a number of preclinical and early clinical trials, polyclonally-expanded Tregs (polyTregs) have demonstrated safety and potential efficacy [4], [5], [6]. However, several theoretical drawbacks exist with polyTreg therapy including the potential for indiscriminate immune suppression [7]. Attention is therefore turning to alloantigen-reactive Tregs (arTregs), which may exhibit enhanced function with less potential for ‘off-target’ immunosuppression.

*Ex vivo*-expansion of freshly isolated Tregs from peripheral blood is generally performed by stimulation of magnetic bead-isolated or flow-sorted cells with anti-CD3/anti-CD28 beads in the presence of recombinant human IL-2 and rapamycin [8]. This leads to non-specific TCR stimulation and proliferation of polyclonally-reactive Tregs (polyTregs). The use of this approach generates significant numbers of CD4^+^FOXP3^+^ cells with a purity that is often improved to over 80% with the use of rapamycin to reduce T effector contaminant proliferation [8]. However, animal studies suggest that high numbers of polyTregs (1:1 to 1:5 Treg to Teff) are required to produce a measurable effect [9].

For example, in humanized mouse models, the adoptive transfer of e*x vivo-*expanded human polyTregs at high numbers can prevent skin, vessel and islet allograft rejection [4], [10], [11], [9]. However, arTregs produced through the co-culture of Tregs with allogeneic DCs or B cells are more effective than polyTregs at preventing this rejection at lower numbers, and demonstrate superior migration and accumulation in the allograft [12], [13], [14], [15]. Use of an enriched population of arTregs may therefore overcome the both the requirement for high cell numbers as well as the off-target specificity of polyTregs.

## 2 Strategies to expand human alloantigen-reactive Tregs *ex vivo*

In healthy individuals, Tregs represent approximately 5–10% of the CD4^+^ T cell population [16], [17], [18], of which only 5–10% are alloantigen-reactive [19], [20]. This low precursor frequency means that cells require extensive *ex vivo*-expansion in order to obtain enough numbers for clinical application. Stimulator populations for arTreg production include peripheral blood mononuclear cells (PBMCs) [12], dendritic cells (DCs) [21] or B cells [13], [22]. Table 1 summarizes the current approaches used in expanding human arTregs.

**Table 1:** Approaches for ex vivo expansion of human alloantigen-reactive Tregs. APC, antigen presenting cell; DC, dendritic cell; PBMC, peripheral blood mononuclear cell; Treg, regulatory T cell; rh, recombinant human.

Starting population	Stimulator	Ratio	Growth factors	Expansion duration	Expansion fold	Reference
CD4^+^CD25^+^Treg isolated by magnetic beads	Donor derived PBMCs	4:1 PBMCs: Tregs	rhIL-2 + rh IL-15	20 days	780	[12]
CD4^+^CD25^+^CD127^-^Treg isolated by magnetic beads	UltraCD40L-activated donor B cells	1:1 B cells: Tregs	rhIL-2 + TGF-β + Sirolimus SRL-7 days only	28 days	~20	[24]
CD4^+^CD25^+^CD127^-^ Treg isolated by FACS	CD40L-activated donor B cells	4:1 B cells: Tregs	rhIL-2	16 days	50–300	[13]
CD4^+^CD25^+^Treg isolated by magnetic beads	Allogeneic mature DCs	1:10 mDCs: Tregs	rhIL-2 + rh IL-15 + Rapamycin	21 days	8.3	[23]
CD4^+^CD25^+^CD127^-^ Treg isolated by FACS	Blood or dermal donor derived mature CD1c^+^ DCs	Not reported	rhIL-2	4–6 weeks	Mean numbers ~2.8 × 10^7^	[14]
CD4^+^CD25^+^Treg isolated by magnetic beads	CD40L-expanded B cell lines	10:1 B cells: Tregs	rhIL-2	2–3 weeks	80–120	[15]
CD4^+^CD25^+^CD127^-^Treg isolated by magnetic beads	Allogeneic monocytes derived DCs	1:10 DCs: Tregs	rhIL-2 + rh IL-15 + Rapamycin	12 days	8	[25]

Irradiated donor-derived PBMCs used as stimulators for Tregs sorted by FACS yield a low expansion rate over a two-week period, although interestingly this expansion improves when Tregs were isolated using magnetic beads (cliniMACS) instead [12]. This suggests that residual antibody binding may subsequently impair Treg expansion. The activation of Tregs requires cell-to-cell contact leading to an immunological synapse, with activation through the TCR and suitable costimulatory signals [23]. Therefore, most protocols rely on the use of purified B cells or DCs as professional antigen presenting cells (APCs) to ensure optimal delivery of signals for Treg activation and proliferation.

The use of B cells for Treg allostimulation requires a preliminary B cell expansion and activation step. As B cells need a CD40/CD40L co-stimulatory signal to proliferate, CD40L-expressing fibroblasts have been used as feeder cells to expand B cells. Immortalized B cell lines from HLA-matched donors have been used to offer a direct expansion of alloantigen Tregs from a readily available allogeneic B cell bank [15]. However, this bank may not cover all HLA-donor/recipient combinations and also has the potential for cellular contamination in the final cell product. Soluble 4-trimer CD40L fusion proteins may represent an alternative to feeder cells and appear to be efficacious in generating arTregs [24].

DCs provide potent allo-stimulatory signals to expand Tregs with a low risk of persistence within the culture, especially when irradiated. Tregs selected using magnetic beads and primed twice by allogenic monocyte-derived DCs (mDCs) cultured with rapamycin, IL-2 and IL-15 have been shown to be functional both *in vitro* and *in vivo*, controlling GVHD in a mouse model [23]. These arTregs expand eight-fold and display a fully demethylated Treg-specific demethylated region (TSDR) with high expression of FOXP3. The most efficient method for generating arTregs remains unclear with no studies having directly compared expansion using alternative stimulatory cell populations from the same donor. This would provide a useful comparison in terms of cellular phenotype and suppressive capability of expanded arTregs.

## 3 Enriching arTregs

**Fig. 1:**
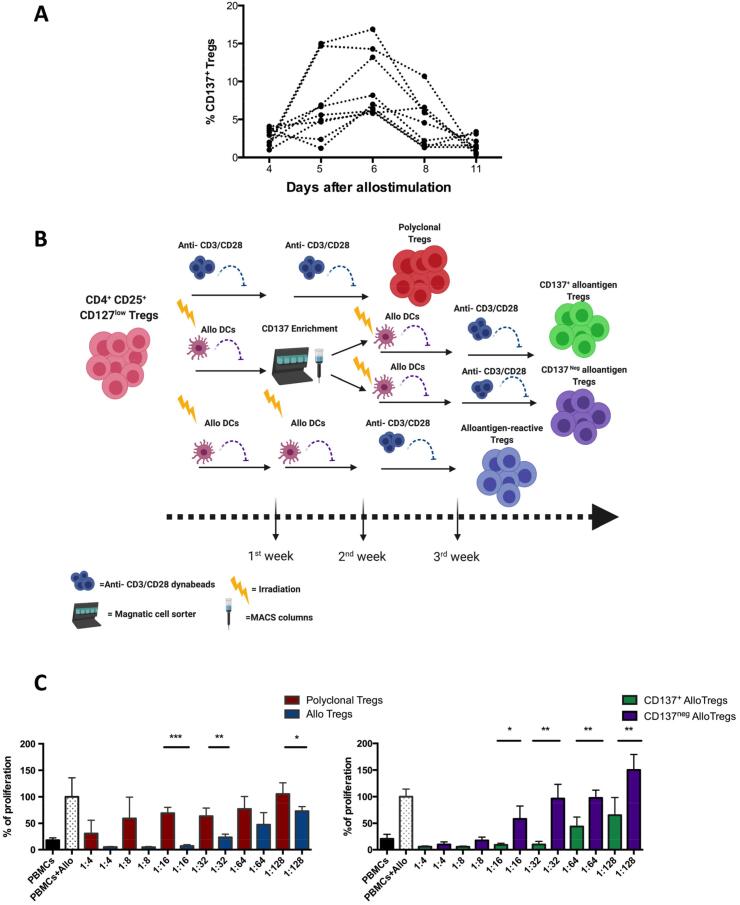
Original dataset. CD137^+^ alloantigen-expanded Tregs (green) are more suppressive than polyclonal Tregs (red), non-enriched Tregs (blue) and CD137^neg^ alloantigen-expanded Tregs (purple). (A) CD137 expression upon alloantigen stimulation of flow-sorted CD4^+^CD25^+^CD127^lo^ human Tregs. Tregs were stimulated with immature dendritic cells (iDCs) at a cell-to-iDC ratio of 4:1. Cells were assessed for CD137 expression by flow cytometry. Data from 9 healthy human donors are shown. (B) Experimental schematic for *in vitro* expansion of polyclonal and alloantigen expanded Tregs created with BioRender.com (C) Suppression assays were performed using 3H-thymidine incorporation; responder cells were stimulated with allogeneic iDCs. Polyclonally expanded, alloantigen-expanded non-CD137 enriched, alloantigen-expanded enriched CD137^+^ and CD137^neg^ Tregs were titrated into the culture. Responders alone were used as a negative control. Responders with alloantigen were used as a positive control. Six days later, thymidine was added to the culture and after 16 h of incubation cells were harvested. Data are represented as mean +/-SD, statistical analysis was performed using unpaired t-tests (*p < 0.05, **p < 0.01, ***p < 0.001). Representative data from one donor out of five is shown.

There is no definitive method to determine which cells within Treg pools are alloreactive. Some have proposed use of Treg-specific activation markers for selection and identification of antigen-reactive Tregs. A number of Treg-specific or Treg-associated activation markers have been described including LAP [26], GARP [27], CD27 [28], CD69 and CD71 [14], and CD137 [29]. For example, CD27^+^ enriched alloantigen-expanded Tregs are significantly more suppressive than CD27^-^ alloantigen Tregs in *in vitro* suppression assays [28]. Another activation marker of human Tregs is CD137 (4-1BB), a member of the tumor necrosis factor receptor (TNFR) family [29]. We have assessed CD137 as a potential marker for identification of arTregs in a series of original experiments on human Tregs *in vitro*. We found CD137 to be upregulated rapidly on Tregs, with levels peaking on day 6 after allostimulation *(*Fig. 1*A)*. Based on this, FACS-sorted Tregs cultured *ex vivo* with allogeneic irradiated immature DCs (iDCs) could then be selected according to CD137 expression on day 6 after allostimulation, followed by further expansion with a combination of alloantigen stimulation and polyclonal anti-CD3/anti-CD28 bead simulation, in order to increase overall yields *(*Fig. 1*B)*. Using an *in vitro* suppression assay, we found CD137^+^-enriched alloantigen-expanded Tregs to be more superior suppressors than CD137^-^ alloantigen Tregs, non-CD137 enriched alloantigen Tregs, or polyclonally-expanded Tregs *(*Fig. 1*C).* This enhanced potency may compensate for the lower final yield of cells at the end of the expansion process. Nevertheless, challenges remain in the feasibility of generating high yields of enriched arTregs at a practical scale for clinical use.

## 4 Direct versus indirect allospecificity

Alloantigen recognition is initiated through three main pathways: direct, indirect, and semi-direct allorecognition [30], [31]. The direct alloresponse occurs when host T cells recognise allogeneic donor APCs presenting allogeneic MHC-peptide complexes [32]. Indirect alloresponses describe the presentation of processed donor-derived peptides by host APCs via their own MHC to host T cells. Semi-direct presentation occurs when host T cells capture intact allogeneic MHC-peptide complexes presented by host APCs, and this pathway is of increasing interest in transplantation [33], [34]. The role of direct allorecognition dominates early after transplantation and can lead to a vigorous immune response. Indirect presentation has been suggested to be the major pathway underlying chronic or late transplant rejection [35], [36]. Attention is turning therefore to arTregs populations with indirect allospecificity to regulate this response.

Indirect allospecificity can be enriched in Tregs through repetitive stimulation with autologous DCs pulsed with donor peptides. Generated arTregs are able to suppress both indirect and direct alloresponses of naïve CD4^+^CD25^-^ T cells *in vitro* [37]. Another approach used in mouse models to generate Tregs with indirect allospecificity is through TCR transduction [38], [39]. In one example, a TCR specific for the H-2K^d^ peptide presented by an MHC class II molecule H2A^b^ was retrovirally transduced into Tregs. These TCR-transduced Tregs (TCR-Tregs), which indirectly recognised allogeneic MHC class II molecules, induced long-term survival of MHC-mismatched heart grafts [39]. Importantly, TCR-Tregs with indirect allospecificity are superior at promoting graft tolerance compared with Tregs with direct allospecificity. This suggests that there should be a focus on the development of arTregs with indirect allospecificity.

## 5 CAR Tregs

Recent advances in chimeric antigen receptor (CAR) technologies have opened the possibility of being able to redirect the specificity of human Tregs as desired [40], [41]. CAR Tregs have shown promise in early experimental models in transplantation [42], [43], [44] and autoimmunity [45], [46]. CAR Tregs specific for MHC-I molecules are superior to polyTregs at preventing xenogeneic GVHD and skin graft rejection in humanized mouse models [42], [43]. However, there are some differences between CAR Tregs and arTregs and challenges that must be overcome before CAR Tregs can be used clinically.

CAR Tregs are produced using a viral vector, in contrast to arTregs which are produced using a simple method of co-culture with donor antigen. Therefore, the safety of CAR Tregs in solid organ transplantation needs to be confirmed, particularly as clinical experience has shown that the adoptive transfer of CAR T cells directed against tumour antigens can result in adverse effects related to cytokine storms and cytotoxicity [47], [48]. While CAR Tregs are unlikely to harbour the same pro-inflammatory potential, care must be taken to ensure that these cells remain stably suppressive or anergic. CAR Tregs are able to inhibit direct allorecognition and consequently acute cellular rejection [42], [44], but their effect on indirect allorecognition-associated responses remains to be understood. Furthermore, alloreactivity may be driven by a broad array of antigens, therefore the broader specificity or arTregs might be preferable. Notably, TCR assessment of arTregs has shown restriction to several clones [24]. This wider clonality allows arTregs to react with multiple donor antigens.

Exhaustion of CAR Tregs is another challenge. Some studies indicate that CAR T cells incorporating the CD28 costimulatory domain have limited *in vivo* expansion and anti-tumor efficacy, which is avoided with the 4-1BB costimulatory domain [49]. For CAR Tregs, studies using second-generation constructs with a CD28 domain have demonstrated excellent suppressive efficacy, with this co-stimulatory molecule appearing to be the most effective of a range tested [50], [51]. Research continues to investigate methods for enhancing function, for example through concomitant regulatory cytokine production, as well as to reduce immunogenicity [52] and improve CAR Treg manufacturing frameworks [53].

## 6 Lessons learned from clinical studies of polyTreg therapy

Over the last few years, a number of early-phase trials have reported their experience in the production and administration of polyTregs (Table 2). Attention is now turning towards alloantigen-specific Treg populations, with some of these beginning to emerge into the clinical arena [54]. Whilst these trials will require their own evaluation of safety and tolerability, there are some lessons learned from polyTreg trials that help develop the path for future development.

**Table 2:** Published studies of expanded Treg adoptive transfer. MACS, magnetic bead sorted; FACS, fluorescence activated cell sorting, bw – body weight.

Year	n	Clinical setting	Phase	Method of Treg Generation	Culture duration (days)	Cell number (per infusion)	Poly/Allo Tregs	Treg Purity	Efficacy	Adverse Events	Ref
2009	2	GVHD after HSCT	I	Autologous MACS-sorted CD4^+^CD25^+^CD127^-^ 2:1 αCD3/αCD28 beads 1000U/mL IL-2	Up to 21	1. 1 × 10^5^cells/ kg bw (single infusion) 2. 3 × 10^6^ cells/ kg bw (three infusions)	Poly	1. 90% 2. 40–90%	1. Improvement in cGVHD, immunosuppression minimised. 2. Transient stabilisation of aGVHD during infusions	Patient 1. None reported Patient 2. Death (from aGVHD after completing course of Tregs)	[63]
2011	23	Prevention of GVHD after HSCT	I	Partially HLA-matched UCB MACS-sorted CD4^+^CD25^+^ 3:1 αCD3/αCD28 beads 300U/mL IL-2	18 ± 1 days	1–30 × 10^5^ cells/kg bw (9/23 single infusion, 14/23 two infusions)	Poly (donor-derived)	31–96% (median 64%)	Similar disease free survival & donor engraftment, 30% reduction in aGVHD cf. historical controls.	No dose-limiting toxicity. Hypertension in 3/23 No ↑ infection/relapse cf historical controls	[64]
2012	10	Newly diagnosed T1DM	I	Autologous FACS-sorted CD4^+^CD25^+^CD127^-^ 1:1 αCD3/αCD28 beads 1000U/mL IL-2	Up to 14	10–20 × 10^6^ Treg/kg bw	Poly	90–97%	Reduction in exogenous insulin requirement and HbA1c after 2 weeks, sustained to 4 months	No serious infections, acute glucose dysregulation or adverse effects	[62]
2015	14	Newly diagnosed T1DM	I	Autologous FACS-sorted CD4^+^CD25^+^CD127^-^ 1:1 αCD3/αCD28 beads 300U/mL IL-2 100 ng/mL rapamycin	14	5 × 10^6^ to 2.6 × 10^9^ cells (single infusion)	Poly	76–97%	No discernable effect upon c-peptide, HbA1c or insulin use	No infusion reactions No infection/malignancy during follow-up 2/16 did not reach release criteria	[65]
2016	10	Living Donor Liver Transplantation	I/IIa	Allo-stimulated PBMC CD80 and CD86 blockade No IL-2/rapamycin	14	0.23–6.4 × 10^6^ Treg /kg bw (single infusion)	Allo	3–45% (median 10%)	7/10 successfully weaned from IS (3/10 - acute rejection)	Alopecia in 1/10 CMV hepatitis in 1/10	[66]
2017	3	Renal transplantation	I	Autologous FACS-sorted CD4^+^CD25^+^CD127^-^ αCD3/αCD28 beads 300U/mL IL-2	14	320 × 10^6^ polyclonal Treg (single infusion)	Poly	>93%	Improvement in inflammation in 2/3, progression to cellular rejection in 1/3	No infusion reactions No patient or graft loss No infection/malignancy during 12 m follow-up	[58]
2018	9	Living Donor Renal Transplantation	I	Autologous MACS-sorted CD4^+^CD25^+^ 4:1 (later 1:1) αCD3/αCD28 1000U/mL IL-2, 1ug/mL TGF-β 100 ng/ml rapamycin	21	0.5–5 × 10^9^ Treg (single infusion)	Poly	>80% (FOXP3 expression)	Subclinical C4d + rejection in 1/9. DSA in 2/9. Recurrence of FSGS in 1/9	No adverse events	[5]
2019	9	Liver transplantation	I/IIa	Autologous MACS-sorted CD4^+^CD25^+^ 2:1 αCD3/αCD28 beads 500U/mL IL-2 100 nM rapamycin	24 – 36	0.5–4.5 × 10^6^ Treg/kg (65–468 × 10^6^ Treg infused)	Poly	61–92%	↓ donor-specific responses in those receiving highest dose of Tregs	No adverse events in low-dose Tregs infusion 1/6 transient pyrexia, leucopenia & graft dysfunction (high-dose)	[57], [61]‬‬‬‬‬‬‬‬‬‬‬‬‬‬‬‬‬‬‬‬‬‬‬‬‬‬‬‬‬‬‬‬‬‬‬‬‬‬‬‬‬‬‬‬‬‬‬‬‬‬‬‬‬‬‬‬‬‬‬‬‬‬‬‬‬‬‬‬‬‬‬‬‬‬‬‬‬‬‬‬‬‬‬‬‬‬‬‬‬‬‬‬‬‬‬‬‬‬‬‬‬‬‬‬‬‬‬‬‬‬‬‬‬‬‬‬‬‬‬‬‬‬‬‬‬‬‬‬‬‬‬‬‬‬‬‬‬‬‬‬‬‬‬‬‬‬‬‬‬‬‬‬‬‬‬‬‬‬‬‬‬‬‬‬‬‬‬‬‬‬‬‬‬‬‬‬‬‬‬‬‬‬‬‬‬‬‬‬‬‬‬‬‬‬‬‬‬‬‬‬‬‬‬‬‬‬‬‬‬‬‬‬‬‬‬‬‬‬‬‬‬‬‬‬‬‬‬‬‬‬‬‬‬‬‬‬‬‬‬‬‬‬‬‬‬‬‬‬‬‬‬‬‬‬‬‬‬‬‬‬‬‬‬‬‬‬‬‬‬‬‬‬‬‬‬‬‬‬‬‬‬‬‬‬‬‬‬‬‬‬‬‬‬‬‬‬‬‬‬‬‬‬‬‬‬‬‬‬‬‬‬‬‬‬‬‬‬‬‬‬‬‬‬‬‬‬‬‬‬‬‬‬‬‬‬‬‬‬‬‬‬‬‬‬‬‬‬‬‬‬‬‬‬‬‬‬‬‬‬‬‬‬‬‬‬‬‬‬‬‬‬‬‬‬‬‬‬‬‬‬‬‬‬‬‬‬‬‬‬‬‬‬‬‬‬‬‬‬‬‬‬‬‬‬‬‬‬‬‬‬‬‬‬‬‬‬‬‬‬‬‬‬‬‬‬
2020	12	Living donor renal transplantation	I	Autologous MACS-sorted CD4^+^CD25^+^ 4:1 αCD3/αCD28 beads 500U/mL IL-2 100 nM rapamycin	36	1 – 10 × 10^6^ Treg/kg bw (single infusion)	Poly	Not yet reported	Not reported	No adverse events	[6], [8]

Early-phase studies of polyTregs focused on the safety and tolerability of Treg infusion in three major clinical areas: new-onset type 1 diabetes mellitus (T1DM), hematopoietic stem cell transplantation, and solid organ transplantation (Table 2). In these studies, Tregs are produced *ex vivo* from PBMCs acquired in sufficient numbers from up to half a litre of blood or by leukopheresis. Leukopheresis avoids the loss of significant red blood volume and has been shown to enable collection of a greater number of PBMCs without causing significant anemia [55], [56]; this may also shorten the duration of Treg culture [57], and may be of use in patients where Treg expansion could be stunted, for example where pre-existing immunosuppression is a factor [58]. This is likely to be of particular use in the settings of hematopoietic stem cell and renal transplantation. Reassuringly, pre-existing immunosuppression and the presence of immune and metabolic abnormalities that may precipitate or accompany end-stage organ dysfunction do not appear to impact upon the ability to generate functional Tregs *ex vivo* in sufficient numbers for clinical administration. This has been reproducibly demonstrated in the setting of uremia (including patients receiving dialysis) [56], [59], [60], cirrhosis [57], [61], after renal and liver transplant [57], [58], and in newly-diagnosed T1DM [62].

## 7 Dosing, tolerability and adverse events

There is considerable variation in the reporting of dosing of Tregs in studies published to date. Infusion of cell products in numbers ranging from 1 × 10^5^ to 7 × 10^7^ cells per kilogram of body weight appears to be well tolerated. Reports of adverse effects, both around the time of infusion and during the follow-up period, are mild and isolated. Certainly any reactions reported are difficult to attribute to the infusion itself, and are not consistently seen in other subjects despite subsequent dose escalation.

Despite concerns regarding ‘off target’ suppressive effects of infused polyTregs, there is no evidence of increased risk of infection or malignancy in the short- to medium-term follow-up periods reported, compared to historical controls. In one study, *in vitro* responses against both polyclonal stimulation with mitogens and more specific stimulation with infection-associated antigens did not demonstrate a reduction in response following infusion compared to beforehand other than that ascribed to pharmacological immunosuppression [5]. In the ONE Study which was a large international consortium assessing multiple immune regulatory cellular therapies, infection rates were demonstrated to be reduced in patients receiving regulatory cell therapies although this might be confounded by the elimination of induction immunosuppression in this arm of the trial [6], [67]. Further data regarding the outcomes of each type of cell therapy assessed in the ONE Study are awaited.

## 8 Assessing efficacy of Treg therapy

Glimpses into efficacy may be gleaned from some of these early-phase studies, though they are intended to focus predominantly on safety and tolerability. The use of polyTregs in the setting of established subclinical inflammation was assessed in one small Phase I study; in two of three patients there was an improvement in histological appearance, whilst in the third there was progression to cellular rejection in the setting of a *de novo* donor specific humoral response which developed immediately prior to, and was not arrested by, the cell infusion [58]. Efficacy assessment is simpler in the autoimmunity setting, where the aim is to arrest or reverse autoimmune disease, which frequently has a detectable clinical marker of activity. A Phase I trial in participants with newly-diagnosed T1DM found a reduction in the requirement for exogenous insulin and an increase in circulating C-peptide levels after two weeks, which was sustained at four months post-infusion compared to matched controls [62]. Two participants did not require exogenous insulin during the follow-up period. A second study failed to replicate this finding using infusion of comparable Treg numbers, although this study enrolled participants at a later time after diagnosis, where the window for intervention may have passed [65].

Only one study, in liver transplantation, has reported on donor-specific alloresponses in recipient PBMC after infusion. Here there was a trend towards a reduction in CD154 upregulation in memory CD8^+^ T cells upon stimulation with surrogate (partially HLA matched) donor PBMC in those receiving a higher, but not a lower dose of Tregs [57]. This trend was not convincingly seen in response to fully mismatched third-party PBMC or CMV antigens. ‬In summary, whilst a demonstration of clinical efficacy is beyond the scope of studies reported to date, expanded polyTregs are functional at time of infusion and there is a indirect data to suggest there may be a subsequent impact upon donor-specific responses with infusion of sufficient numbers of cells.

## 9 Survival and stability of infused Tregs

An elevation in total circulating Treg numbers or proportions has been reported in both immunosuppressed and non-immunosuppressed patients which persists beyond the immediate post-infusion period [5], [6], [57], [62]. In one early study in T1DM, an elevation in the Treg proportion was seen for up to four months following infusion [62]. In the setting of renal transplantation, an elevation in Treg numbers has been observed for one year after infusion; a phenomenon not seen in historical controls receiving the same immunosuppressive protocol [5]. In a study in liver transplantation, there was an increase in circulating Tregs by three days post-infusion, persisting for a month, not seen in those receiving a lower infusion dose [57]. The duration of elevation appears to correlate with the number of Tregs infused. However, this does not provide information on whether these represent the original infused Tregs or reactive expansion of a ‘native’ Treg population.

Identification of infused Tregs to facilitate evaluation of population kinetics after cell infusion is informative. In one study in bone marrow transplantation, HLA-mismatched, cord-blood origin Tregs were detectable in peripheral blood for up to 14 days [64]. Deuterium-labelled infused Tregs peaked in the circulating Treg pool within seven to fourteen days, but continued to remain detectable for over a year in some participants with T1DM [65]. Reassuringly deuterium labelling could not be detected in circulating non-Treg populations, suggesting there had not been any major transdifferentiation of infused Tregs into an effector T cell phenotype. A similar strategy uses gadolinium to label cells for detection [68]. It is feasible that pharmacological immunosuppression could impede the survival of infused Tregs, however a small study in renal transplant recipients on established calcineurin inhibitor-based immunosuppression post-transplantation did not suggest this to be the case, with similar kinetics to matched patients receiving an infusion for new-onset diabetes [58]. Whilst calcineurin inhibition has been associated with destabilization of Tregs in non-human primate models [69], deuterium labelling was only seen within the Treg gate throughout follow-up.

## 10 Trials of arTregs

To date, only a single clinical study, in the setting of liver transplantation, has tested arTregs, generated using donor peripheral blood leucocytes cocultured with unselected recipient splenocytes in the setting of costimulatory blockade [66]. The protocol generated CD4^+^CD127^low^FOXP3^+^ Tregs with a relatively low purity within the CD4^+^ population averaging <25%. The infused cell product was heterogeneous, with expanded CD19^+^ and CD8^+^ cells also infused. Participants underwent a regimen of splenectomy and cyclophosphamide administration prior to Treg infusion. Acute rejection upon per-protocol weaning of immunosuppression appeared to be limited to those with a history of autoimmune liver disease, with the remaining participants all demonstrating stable graft function up to three years after drug discontinuation. *In vitro* assays indicated that PBMCs from many participants had reduced proliferative activity in response to donor stimulation to a greater degree than that seen with third-party stimuli, even after drug discontinuation. Interestingly, the immunological picture did not clearly correspond to the clinical one, with a degree of donor-specific hyporesponsiveness seen even in those who developed acute rejection. However, this study was limited by the lack of a prospectively recruited control arm and the inherent advantage of liver transplants being permissive to immunosuppression withdrawal. A number of early-phase clinical trials of arTregs in the setting of solid organ transplantation are currently ongoing and will report in the coming years (Table 3).

**Table 3:** Ongoing trials of polyclonal and alloreactive Tregs. Clinicaltrials.gov* and EudraCT** were searched using the keywords “regulatory T cells” or “Tregs” in the disease area “transplantation”. Results were filtered by studies with status “ongoing”, “recruiting”, “active, not yet recruiting” and “enrolling by invitation”. Search date: 3 Jan 2020.

Trial ID	Phase	Tregs	Dose	Design	Setting	n (Treg arm)	Start Date	Primary Completion Date	Status
NCT03284242*	N/A	Polyclonal	Not specified	Non-randomised	Renal transplantation with everolimus immunosuppression	12	March 2019	December 2019	Recruiting
NCT02091232*	I	Alloreactive	Not specified	Non-randomised	Living donor renal transplantation	8	May 2014	March 2018	Active, Not Recruiting
NCT03943238*	I	Not specified	Not specified	Non-randomised	Living donor renal transplantation, with donor BM infusion	22	June 2019	August 2024	Not Yet Recruiting
NCT03577431*	I/IIa	Alloreactive	2.5–500 × 10^6^ cells (single dose)	Non-randomised	Liver transplantation, with subsequent immunosuppression withdrawal	9	March 2019	March 2023	Recruiting
NCT02474199*	I/IIa	Alloreactive	400 × 10^6^ cells (single dose)	Non-randomised	Living donor liver transplantation, with subsequent immunosuppression withdrawal	13	June 2016	December 2019	Active, Not Recruiting
NCT03867617* 2018–003142-16**	I/IIa	Not specified	10 × 10^6^ cells (single dose)	Non-randomised	Renal transplantation, with donor BM infusion & toclizumab	12	August 2019	April 2023	Recruiting
NCT02711826*	I/IIa	Polyclonal	>300 × 10^6^ cells (single dose)	Open-label, randomised	Renal transplantation with subclinical inflammation on protocol biopsy > 5 m post transplant	15	May 2016	October 2021	Recruiting
2017–001421-41**	IIb	Polyclonal	5–10 × 10^6^ cells/kg (single dose)	Open-label, randomised	Living donor renal transplant with immunosuppression minimisation	68	Feb 2018	Feb 2022	Recruiting

## 11 Outstanding questions and future directions

The expanding body of literature around early-phase trials of polyTregs demonstrates that acquisition, expansion and re-infusion of Treg is both feasible and well tolerated. This provides confidence when moving towards trials of arTregs. However, in addition to the outstanding questions raised so far there are a number of other considerations which will need to be addressed in future work, prior to the use of arTregs clinically.

## 12 Safety

The motivation for the development arTregs is to reduce off-target immunosuppression; however it is possible that enhanced local bystander suppression due to activation of large numbers of arTregs could lead to an increased risk of local infection or reactivation of latent viruses present within the graft, such as BK virus in renal allografts [70].

## 13 Immunosuppression and timing of infusion

The impact of immunosuppressive drugs including tacrolimus, mycophenolate and methylprednisolone on the therapeutic efficacy of infused Tregs has previously been assessed in a humanized mouse model [71]. The viability and proliferative capacity of Tregs were reduced in a dose dependent manner by these drugs. The only immunosuppressant consistently demonstrated to potentiate Treg expansion and survival is rapamycin. However, poor patient tolerance of rapamycin has led to a search for alternative strategies to synergize with Treg infusion. For example, low-dose IL-2 may aid in restoring Treg numbers and function after hematopoietic stem cell transplantation without concurrent expansion of effector T cell populations, with an improvement in GVHD in around half of patients [72], [73]. In addition, IL-2 treatment in a mouse skin transplant model has been shown preferentially enhance the proliferation of infused arTregs in an antigen-dependent manner [74]. Combination therapy with low-dose IL-2 and arTregs is therefore an appealing approach.

The optimal time for Treg infusion to maximize efficacy is unclear. While early infusion would be more likely to promote tolerance development prior to the establishment of immunological memory, the persistence of induction agents could impair the survival or proliferation of Tregs. Transient host leukodepletion alongside arTregs infusion promotes allograft survival beyond that of arTregs infusion alone in pre-clinical models [75]. However, a number of lymphodepleting agents in clinical use can be detected for weeks post-infusion. Other non-depleting agents widely used at induction include IL-2 receptor antagonists, which are likely to prevent the IL-2 signalling critical for Treg survival [76]. These considerations mean that cell infusion should probably be delayed until these agents have cleared.

PolyTregs can be generated and stored prior to transplantation using recipient PBMC alone, however arTregs require donor-specific stimulation and this creates a logistical challenge. The use of arTregs clinically would therefore be limited to living donor transplantation, where the donor is identified and available prior to transplant. Alternatively, infusion of arTregs may be delayed to allow for the time required for allogenic stimulation and expansion.

## 14 Conclusions

After nearly three decades of preclinical research into Treg biology, we are beginning to see the progression of Treg therapy through clinical trials. Early phase trials of polyTregs have provided valuable information on cell therapy safety in transplantation as well as some very promising glimpses of efficacy. In the future, more directed allospecific Treg therapy is likely to be preferable, both in terms of off-target and immunological effects. This may be in the form of arTregs or engineered Treg products such as CAR or TCR-transgenic Tregs. Preclinical studies in both human and animal models have provided insight into useful strategies to generate these cellular therapies. Early phase trials of arTregs and CAR Tregs are currently underway, with the coming years likely to be both exciting and enlightening with respect to the future of allospecific Treg therapies in transplantation.

Funding

AA is funded 10.13039/501100004054by King Abdulaziz University, Saudi Arabia. MB receives research funding from the 10.13039/501100000296British Skin Foundation, 10.13039/501100000769University of Oxford and Oxford Hospitals Charitable Trust. JH receives funding from 10.13039/501100000291Kidney Research UK. FI receives funding from the 10.13039/100010269Wellcome Trust and the 10.13039/501100000265Medical Research Council. We acknowledge funding from the EU Horizon 2020 Research and Innovation Programme under grant agreement 825392 (RESHAPE).
